# Awareness of knowledge and practice regarding physical activity: A population-based prospective, observational study among students in Nanjing, China

**DOI:** 10.1371/journal.pone.0179518

**Published:** 2017-06-16

**Authors:** Fei Xu, Xiaorong Wang, Dandan Xiang, Zhiyong Wang, Qing Ye, Robert S. Ware

**Affiliations:** 1 Nanjing Municipal Center for Disease Control and Prevention, Nanjing, China; 2 Department of Epidemiology, Nanjing Medical University School of Public Health, Nanjing, China; 3 Nanjing Children’s Hospital Affiliated to Nanjing Medical University, Nanjing, China; 4 Menzies Health Institute Queensland, Griffith University, Brisbane, Queensland, Australia; Old Dominion University, UNITED STATES

## Abstract

**Background:**

Physical activity (PA) promotion has proven effectiveness in preventing childhood obesity. Increasing children’s health knowledge is the most frequently used approach in PA intervention programs targeting childhood obesity prevention. However, little is known about the specific association between the change in a child’s knowledge awareness and their PA practice.

**Methods:**

A one-year follow-up study was conducted among primary and junior high school students in Nanjing, China. At baseline students’ knowledge of healthy behavior, and their PA levels, were assessed. Students who were unaware of the association between PA and obesity were followed for one academic year. After nine-months their knowledge and PA levels were re-measured using the same validated questionnaire. Mixed effects regression models were used to estimate the relationship between awareness of knowledge about the link between PA and obesity and PA changes.

**Results:**

Of the 1899 students who were unaware of the association between PA and obesity at baseline, 1859 (follow-up rate = 97.9%) were successfully followed-up. After nine months 1318 (70.9%) participants had become aware of PA-obesity association. Compared to their counterparts who remained unaware, students who became aware of the PA-obesity association were more likely to increase both the frequency (odds ratio (OR) = 1.34, 95%CI = 1.09, 1.64) and duration (OR = 1.34, 95%CI = 1.09, 1.65) of PA, after adjusting for potentially confounding variables.

**Conclusion:**

Becoming aware of the known link between PA and obesity led to positive behavior modification regarding PA in this cohort of Chinese students. This is of particular importance that knowledge disimination and health education may be a useful approach for population-based physical activity promotion aiming at childhood obesity prevention in China.

## Introduction

Childhood obesity is a major public health concern globally due to its continuously increasing prevalence worldwide, including within China [1–3]. Obesity in childhood not only has adverse effects on children's physical and mental health, but also predicts adulthood obesity and influences morbidity and mortality in adult life [4–6]. Physical inactivity is one of the major contributors to the alarming increase in childhood obesity [7–10]. As a consequence increased physical activity (PA) promotion is urgently needed for childhood obesity prevention.

Previous studies have documented that school-based PA interventions are effective in preventing obesity among school students in different cultural, social and educational environments [9–13]. Further understanding regarding the factors that influence PA engagement is of great importance, as they will allow tailoring of interventions to specific groups of school children. PA engagement has been shown to be influenced by a number of factors, including psychological (including awareness of knowledge), physiological, socio-cultural, socio-economic and environmental [14]. Among those factors, the association between awareness of knowledge regarding the known association between PA and body weight, and PA engagement has rarely been investigated. According to the ‘Knowledge, Attitude and Practice’ (KAP) model, people may modify their health and lifestyle behaviors if they have specific knowledge about how their behaviors can increase their disease risk [15]. Although knowledge of the benefits of PA has often been included as s component of PA promotion campaigns aimed at children, very few studies have investigated the influence of knowledge on children’s PA practice [16–20].

We hypothesized that becoming aware of the knowledge that PA and body weight are correlated will be positively associated with PA engagement among school children. The aim of this study was to examine the association between change in knowledge and PA modification among school students in Nanjing, China.

## Methods

### Study design and participants

This prospective study was conducted over one academic school year among primary and junior high school students in Nanjing, China. Nanjing is a large city in eastern China with a population of approximately eight million, and is administratively divided into eight urban and five suburban districts. There were two questionnaire surveys implemented. The baseline survey was conducted in September 2013, and the follow-up survey in June 2014. Both surveys collected information on knowledge regarding health behaviors and chronic disease, and PA engagement.

Participants for the baseline survey were children in Grade 4 and Grade 7 (aged approximately 9 and 12 years respectively) from primary and junior high schools within the urban districts of Nanjing. They were initially recruited as part of a large scale obesity prevention program through a multi-stage sampling approach. Four primary and two junior high schools were randomly selected from each of the eight urban districts (32 primary and 16 junior high schools in total). All 4th and 7th graders from the selected schools were invited to participate in the study, resulting in 10447 students who were eligible for the baseline survey.

There were 10091 students (96.6% of eligible) successfully recruited. Students had information on their demographic and social characteristics assessed via questionnaires administered by trained health professionals. The collected information included student’s knowledge about PA and obesity, and the association between PA and obesity [21, 22]. Participant’s anthropometric measurements were recorded by registered doctors within the classroom with the assistance of a class teacher. The question "Do you know that doing regular sufficient PA can help you keep your body losing weight and in good shape (prolonged physical inactivity can have you become obese)?" was used to assess whether participants knew about the influence of PA on obesity (PA-Obesity knowledge). Of the 10 091 respondents at baseline, 1899 (18.8%) students were identified as being unaware of PA-Obesity knowledge. These 1899 students were eligible to be included in the present study, and were re-approached at the follow-up survey. This study was approved by the academic and ethical committee of Nanjing Municipal Center for Disease Control and Prevention (Nanjing CDC). The signed informed consents for this study were obtained from parents/guardians and the schools prior to the baseline survey.

### Study variables

#### Outcome variable

Students' PA level was assessed with a validated item-specific questionnaire[23]. Students were asked to report, item by item, how many occasions (frequency) they engaged in after-school PA in the last 7 days, and the length of their engagement on each occasion (duration). This allowed the calculation of the total amount of time spent in PA in the past 7 days for each participant. This calculation was performed separately at baseline and follow-up. As well as being analyzed as interval variables, two dichotomous outcome measures were developed based on changes in PA frequency and total time (follow-up—baseline): (1) "PA frequency” (PA-F) and (2) "PA time” (PA-T). Both PA-F and PA-T were coded as binary variables (increased/did not increase).

#### Exposure variable

All students were asked the same question regarding their PA-Obesity knowledge at the baseline and follow-up surveys ("Do you know that doing regular sufficient PA can help you keep your body losing weight and in good shape (prolonged physical inactivity can have you become obese)?"). The question was asked before PA was assessed. Students who became aware of the association between PA and obesity during the study period were classified as "became aware", while those who didn’t were classified as “remain unaware”.

#### Covariates

Anthropometric measures were taken twice at each data collection point and the mean of the two readings was used. Weight and height were measured to the nearest 0.1 kilograms and to the nearest 0.01 meter, respectively. Body weight status (excess body weight) was assessed based on each student's body mass index (BMI) which was calculated as weight in kilograms divided by the square of the height in meters according to the age- and sex-specific recommendation for Chinese children by the Group of China Obesity Task Force [24]. The key demographic and social characteristics students’ age, gender, grade, school, and parents’ educational attainment (<high school education/≥high school education) were collected via questionnaire.

### Data analysis

Summary statistics are presented as mean (standard deviation) for continuous variables and as frequency (percentage) for categorical variables. The associations between age, sex, grade, BMI and overweight/obesity status and the binary variable follow-up status were investigated univariably using mixed-effects logistic regression models, with school included as the random effect. The associations between student characteristics and changes in knowledge awareness were investigated using mixed-effects logistic regression models, with school included as the random effect. The associations between change in knowledge awareness (became aware/remained unaware) and PA modification were examined using mixed-effects linear regression models, with change in PA-F and PA-T as the outcome variables and school included as a random effect. Associations were investigated overall, and after stratification for grade and sex. Effect estimates are presented as mean difference (MD) and 95% confidence interval (CI). The association between change in knowledge awareness (became aware/remained unaware) and the binary outcomes increase in PA-F (yes/no) and increase in PA-T (yes/no) was examined using mixed effects logistic regression with school included as a random effect. This association was first investigated univariably, and then in multivariable models with students' age, grade, gender, overweight/obesity and parental education included as covariables. In all mixed-effects models school was included as a random effect to account for potential school-level clustering effects. Effects estimates from logistic regression models are presented as odds ratio (OR) and 95%CI. All analyses were conducted using SAS Version 9.4 software (SAS Institute, Cary, NC).

## Results

Of the 1899 students who were not aware of the association between PA and obesity at baseline, 1859 (97.9%) were successfully followed-up at the end of the academic year. There was no significant differences between those lost and followed in terms of age, gender, grade, and body weight status (Table 1). At study completion, 1318 (70.9%) participants had become aware of PA-obesity knowledge while 541 (29.1%) remained unaware. Table 2 presents the association between participants' characteristics and PA-obesity knowledge at study end. Participants were more likely to become aware of the knowledge if they were older, and heavier at baseline.

**Table 1:** Comparison of selected baseline characteristics between those lost and followed-up.

Characteristic	Lost (n = 40)	Follow-up (n = 1859)	P-value^a^
Mean age (SD)	10.53±1.54	10.61±1.56	0.89
BMI(kg/m^2^)	20.49±4.15	18.53±3.64	0.12
Grade (%)			
4th	21(52.5)	891(47.9)	0.80
7th	19(47.5)	968(52.1)	
Gender(%)			
Boys	21(52.5)	962(51.7)	0.97
Girls	19(47.5)	897(48.3)	
Overweight/obesity (%)			
No	23(57.5)	1401(75.4)	0.24
Yes	17(42.5)	458(24.6)	

^a^ Estimated based on univariate mixed-effects logistic regression models with school included as the random effect.

**Table 2:** Selected baseline characteristics of 1859 participants by end-point status of knowledge in Nanjing, China.

Characteristic	Overall (n = 1859)	Changes in knowledge awareness at follow-up survey		OR(95%CI) ^a^	P-value^a^
		Become aware (n = 1318)	Remain unaware (n = 541)		
Mean age (SD)	10.61±1.56	10.51 ± 1.56	10.85±1.54	0.88(0.81,0.95)	<0.01
BMI(kg/m^2^)	18.53±3.64	18.64±3.67	18.25±3.54	1.04(1.01,1.07)	<0.01
Grade(%)					
4th	891(47.9)	680(51.6)	211(39.0)	1	<0.01
7th	968(52.1)	638(48.4)	330(61.0)	0.60(0.47,0.77)	
Gender(%)					
Boys	962(51.7)	673(51.1)	289(53.4)	1	0.27
Girls	897(48.3)	645(48.9)	252(46.6)	1.12(0.92,1.37)	
Parents' education(%)					
<High school education	173(9.3)	127(9.6)	46(8.5)	1	0.51
≥High school education	1686(90.7)	1191(90.4)	495(91.5)	0.89(0.62,1.27)	
Overweight/obesity (%)					
No	1401(75.4)	971(73.7)	430(79.5)	1	<0.01
Yes	458(24.6)	347(26.3)	111(20.5)	1.40(1.09,1.79)	

^a^ Estimated based on univariate mixed-effects logistic regression models with school included as the random effect.

The associations between change in knowledge awareness and PA-F and PA-T at study end are displayed in Table 3. Compared to their counterparts who remained unaware of the link between PA and obesity at study end, these students who became aware significantly increased both their overall PA-F (MD = 2.5 sessions/week; 95%CI = 0.6, 4.5; p<0.01) and their PA-T (MD = 12.7 minutes/day; 95%CI = 2.7, 22.8; p = 0.01). After stratification, statistically significant differences was observed for both PA-F and PA-T for both boys and the 7^th^ graders, while a statistically significant difference was observed only for PA-F among the 4^th^ graders.

**Table 3:** The associations between change in knowledge and modification of PA-F and PA-T at study end, overall and stratified by grade and gender among urban students in Nanjing, China.

Awareness at study end	Participants number	PA Frequency (per week) mean (SD)			Regression Coefficients (β and 95%CI) ^a^				PA Time (min/day) mean (SD)			Regression Coefficients (β and 95%CI) ^a^		
		Baseline	Follow-up	Changes ^a^	MD^b^		95%CI	P	Baseline	Follow-up	Changes ^a^	MD^b^	95%CI	P
Overall	1859													
Not know yet	541	18.10±15.68	19.59±16.14	1.49±17.63	ref.				78.30±81.88	72.49±75.51	-5.81±88.38	ref.		
Become aware	1318	19.98±18.09	24.16±17.17	4.18±19.95	2.51		0.56,4.46	<0.001	83.81±95.51	90.71±84.43	6.90±103.87	12.73	2.66,22.79	0.01
4th	891													
Not know yet	211	21.99±18.58	23.68±19.10	1.69±21.19	ref.				96.92±97.44	90.92±83.48	-6.01±104.59	ref.		
Become aware	680	23.84±20.81	28.05±19.10	4.20±23.54	2.36		-1.21,5.93	0.005	102.75±112.25	110.61±94.23	7.86±124.43	14.54	-4.07,33.15	0.13
7th	968													
Not know yet	330	15.62±12.93	16.97±13.31	1.35±14.96	Ref.				66.4±67.67	60.71±67.47	-5.69±76.40	ref.		
Become aware	638	15.87±13.50	20.02±13.70	4.15±15.23	2.66		0.64,4.68	0.01	63.64±68.10	69.51±66.32	5.87±76.16	11.81	1.62,22.01	0.02
Boys	962													
Not know yet	289	18.99±15.96	20.82±17.41	1.83±18.48	ref.				85.33±88.77	78.11±76.66	-7.22±90.07	ref.		
Become aware	673	20.57±19.16	25.36±18.23	4.78±21.68	2.94		0.03,5.86	0.048	88.30±97.88	98.84±85.41	10.54±108.29	18.17	3.68,32.65	0.01
Girls	897													
Not know yet	252	17.08±15.32	18.17±14.45	1.09±16.64	ref.				70.25±72.51	66.04±73.79	-4.20±86.54	ref.		
Become aware	645	19.37±16.90	22.92±15.92	3.54±17.95	2.36		-0.23,4.94	0.07	79.13±92.82	82.23±82.6	3.10±98.99	6.77	-7.34,20.88	0.35

^a^ Changes = Follow-up-Baseline.

^b^ MD (mean difference) and 95%CI estimated multivariably with age, gender, grade, overweight/obesity, and parents' education included as co-variables and with school included as a random effect to account for school-level clustering effects.

Table 4 shows the association between change in knowledge awareness and the likelihood of increasing PA-F and PA-T. After adjustment for potential confounders, students who became aware of the PA-Obesity knowledge had 1.34 (95%CI = 1.09, 1.64) and 1.34 (95%CI = 1.09, 1.65) times greater odds of having their PA-F and PA-T improved relative to their counterparts who remained unaware of the knowledge at study completion. When data were separately analyzed by grade and gender, the associations remained statistically significant among the seventh graders for both PA-F and PA-T and among boys for PA-T and girls for PA-F only.

**Table 4:** The relationship between awareness of knowledge and the likelihood of increase in PA-F and PA-T among urban students in Nanjing, China.

Awareness at study end	Participants number	PA frequency Increased			PA time Elevated		
		Yes	Model 1^a^	Model 2^b^	Yes	Model 1^a^	Model 2^b^
		n (%)	OR(95% CI)	OR(95% CI)	n (%)	OR(95% CI)	OR(95% CI) ^a^
Overall	1859						
Not know yet	541	280(51.8)	1	1	252(46.6)	1	1
Become aware	1318	801(60.8)	1.34(1.09,1.65)	1.34(1.09,1.64)	717(54.4)	1.33(1.08,1.63)	1.34(1.09,1.65)
4th	891						
Not know yet	211	117(55.5)	1	1	107(50.7)	1	1
Become aware	680	408(60.0)	1.23(0.90,1.68)	1.25(0.91,1.72)	376(55.3)	1.21(0.88,1.66)	1.25(0.92,1.73)
7th	968						
Not know yet	330	163(49.4)	1	1	145(43.9)	1	1
Become aware	638	393(61.6)	1.42(1.08,1.86)	1.40(1.07,1.85)	341(53.4)	1.41(1.07,1.85)	1.41(1.07,1.85)
Boys	962						
Not know yet	289	152(52.6)	1	1	134(46.4)	1	1
Become aware	673	409(60.8)	1.20(0.90,1.59)	1.22(0.92,1.62)	377(56.0)	1.44(1.09,1.92)	1.46(1.09,1.94)
Girls	897						
Not know yet	252	128(50.8)	1	1	118(46.8)	1	1
Become aware	645	392(60.8)	1.53(1.14,2.06)	1.53(1.13,2.05)	340(52.7)	1.24(0.92,1.67)	1.24(0.92,1.67)

^a^ Model 1: OR and 95%CI estimated univariably with school included as a random effect to account for school-level clustering effects.

^b^ Model 2: OR and 95%CI estimated multivariably with age, gender, grade, overweight/obesity, and parents' education included as co-variables and with school included as a random effect to account for school-level clustering effects

## Discussion

This population-based prospective study examined the relationship between change in knowledge and behavior modification regarding physical activity among urban school students in Nanjing, China, and found that for students who became aware of the association between physical activity and body weight became more physicallly active. This encouraging finding regarding knowledge and PA improvement was particularly prominent in boys and children in the 7^th^ grade.

According to the KAP model, being aware of knowledge can help change related attitudes and lead to the corresponding behavior modification [15]. This model has been supported by previous health behavior-related studies, in topics such as smoking [25], eating [26] and weight control [27]. Our study provided further evidence to this model in this context of better knowledge understanding leading to better PA engagement.

There are very few population-based surveys that have explored the relationship between awareness of knowledge and PA enagement [16–20]. All previous studies have had a cross-sectional design and only three recruited chidlren as participants [16–20]. Of the three studies conducted among children, two reported a positive relationship between awareness of knowledge [18, 19] and PA engagement, while one documented no association between knowledge and PA [20]. In contrast with these previous studies, our study was a population-based prospective study with follow-up conducted 9 months after baseline, a large sample size (n = 1859), and both PA frequency and duration assessed using a validated questionnaire. The nature of our prospective study design allowed us to examine the association between changes in knowlege and modifications of PA engagement, adding a stronger and more robust layer of evidence to this research topic.

The proportion of Chinese adolescents who are physically inactive is higher than that in many other countries in the world [28]. This may be because Chinese students spend much more time in sitting for curriculum study purposes relative to their counterparts in Western countries [29], and consequently have less free-time to take part in PA after school. Even under this special cultural, social and educational context, the positive findings from our study strongly suggest that students who do not know of the link between PA and body weight will engage in more PA when they became aware of this knowledge. From the public health viewpoint, this is of particular importance and there are clear implications for population-based PA promotion campaigns and obesity prevention programs among students in China.

This study has several particular strengths. First, this is not only the first study to consider the relationship between awareness of knowledge and PA practice in China, but it has revealed a positive association between knowledge awareness and behavior modification among students, which added more evidences to surpport the KAP model. Second, the sample size was relatively large compared with previous studies, and sufficiently powered to identify significant between-group differences. And, third, the instrument used to gather information on PA was an item-specifc questionnaire deveoloped specially for Chinese students that shows good validaty and reliability [24].

There are at least four study limitations that need to be mentioned. First, PA level was self-reported, which might produce recall bias [30], although the questionnaire used has demonstrated validity and reliability, and strict quality control approaches were introduced to all field surveys inclduing having class teachers onsite when questionnare surveys were conducted. Second, the assoication between PA and body weight is more complex than the simple question asked of students implies. Third, parents play important role for childrens’ behavior, but only educational attainment was considered as a covariable in the analysis when adjusting for potential parental influence on childrens’ behavior. Fourth, any extrapolation of the findings should be prudent as that a positive association between awareness of knowledge and improvement of PA engagement was examined from a local student population and changes in knowldge accounted for only 4.0% varaition of changes in PA improvement.

In conclusion, knowlede awareness can lead to positive behavior modification regarding PA among Chinese students. It suggested that health education and knowledge dissemination are important in PA promotion campaigns among school students. This is of particular public health implication that knowledge disimination and health education may be a useful approach for population-based physical activity promotion aiming at childhood obesity prevention in China. Future research should explore potential influence of knowledge on physical activity engagement and further consider sustainable knowledge education program of physical activity for childhood obesity prevention under currently existing educational system in a specific society.
